# Postoperative radiological assessment of the mastoid facial canal in cochlear implant patients in correlation with facial nerve stimulation

**DOI:** 10.1007/s00330-021-08128-w

**Published:** 2021-07-05

**Authors:** Iris Burck, Rania A. Helal, Nagy N. N. Naguib, Nour-Eldin A. Nour-Eldin, Jan-Erik Scholtz, Simon Martin, Martin Leinung, Silke Helbig, Timo Stöver, Annette Lehn, Thomas J. Vogl

**Affiliations:** 1 Department of Diagnostic and Interventional Radiology, University Hospital Frankfurt, Theodor-Stern-Kai 7, 60590 Frankfurt, Germany; 2 Department of Radiology, AMEOS Clinic Halberstadt, Halberstadt, Sachsen-Anhalt Germany; 3 Department of Diagnostic and Interventional Radiology, Alexandria University Hospital, Alexandria University, Alexandria, Egypt; 4 Department of Diagnostic and Interventional Radiology, Cairo University Hospital, Cairo University, Cairo, Egypt; 5 Department of Otorhinolaryngology, University Hospital Frankfurt, Frankfurt, Germany; 6 Department of Biostatistics and Mathematical Modeling, University Hospital Frankfurt, Frankfurt, Germany

**Keywords:** Cochlear implantation, Facial nerve, Electric stimulation, Cone-beam computed tomography

## Abstract

**Objectives:**

To correlate the radiological assessment of the mastoid facial canal in postoperative cochlear implant (CI) cone-beam CT (CBCT) and other possible contributing clinical or implant-related factors with postoperative facial nerve stimulation (FNS) occurrence.

**Methods:**

Two experienced radiologists evaluated retrospectively 215 postoperative post-CI CBCT examinations. The mastoid facial canal diameter, wall thickness, distance between the electrode cable and mastoid facial canal, and facial-chorda tympani angle were assessed. Additionally, the intracochlear position and the insertion angle and depth of electrodes were evaluated. Clinical data were analyzed for postoperative FNS within 1.5-year follow-up, CI type, onset, and causes for hearing loss such as otosclerosis, meningitis, and history of previous ear surgeries. Postoperative FNS was correlated with the measurements and clinical data using logistic regression.

**Results:**

Within the study population (mean age: 56 ± 18 years), ten patients presented with FNS. The correlations between FNS and facial canal diameter (*p* = 0.09), wall thickness (*p* = 0.27), distance to CI cable (*p* = 0.44), and angle with chorda tympani (*p* = 0.75) were statistically non-significant. There were statistical significances for previous history of meningitis/encephalitis (*p* = 0.001), extracochlear-electrode-contacts (*p *= 0.002), scala-vestibuli position (*p =* 0.02), younger patients’ age (*p* = 0.03), lateral-wall-electrode type (*p* = 0.04), and early/childhood onset hearing loss (*p* = 0.04). Histories of meningitis/encephalitis and extracochlear-electrode-contacts were included in the first two steps of the multivariate logistic regression.

**Conclusion:**

The mastoid-facial canal radiological assessment and the positional relationship with the CI electrode provide no predictor of postoperative FNS. Histories of meningitis/encephalitis and extracochlear-electrode-contacts are important risk factors.

**Key Points:**

*• Post-operative radiological assessment of the mastoid facial canal and the positional relationship with the CI electrode provide no predictor of post-cochlear implant facial nerve stimulation.*

*• Radiological detection of extracochlear electrode contacts and the previous clinical history of meningitis/encephalitis are two important risk factors for postoperative facial nerve stimulation in cochlear implant patients.*

*• The presence of scala vestibuli electrode insertion as well as the lateral wall electrode type, the younger patient’s age, and early onset of SNHL can play important role in the prediction of post-cochlear implant facial nerve stimulation.*

## Introduction

Cochlear implant (CI) surgery is considered to be a generally safe method for the treatment of severe sensorineural hearing disorders with a low complication rate [1]. However, as the facial recess lies in the insertion plane of the CI, the facial nerve may be stimulated by the device presenting as an abnormal sensation or blinking on the affected side [2]. This electrical irritation of the facial nerve after CI surgery is called facial nerve stimulation (FNS). Postoperative incidence for FNS ranges between 0.9 and 14.9%. To eliminate this side effect, reprogramming of the CI or re-surgery may be required [3, 4].

Postoperative FNS occurrence was explained differently by many investigators. Prior studies suggested that the electric current may bypass the basal turn of the cochlea and stimulate the nearby labyrinthine segment of the facial nerve, especially in cases of decreased bony impedance as in otosclerosis, temporal bone fractures, or post-meningitic labyrinthitis ossificans [4, 5]. Others suggested an association between FNS and young age, cochlea-vestibular anomalies, type of CI device, and higher stimulation currents required in patients with long-term auditory deprivation. However, FNS may occur despite the absence of all of them [3, 6–8].

Only few studies evaluated the positional relationship between the labyrinthine facial nerve and the basal turn of the cochlea regarding the occurrence of FNS. These studies showed a positive correlation between the occurrence of FNS and a short distance or bony dehiscence between the facial nerve and the cochlea [2, 9].

Since the electrode cable and in some cases presence of extracochlear electrodes may have a direct relation to the mastoid facial canal, the mastoid facial nerve may be implicated in cases with FNS; however, to date, no studies exist assessing the mastoid facial nerve radiologically in cases with FNS.

The purpose of this study was to correlate the postoperative radiological assessment of the mastoid facial canal in the facial recess region and its relation to the cochlear implant (including the distance between the electrode cable and the mastoid part of the facial canal, the facial-chorda tympani angle, the diameter, and the wall thickness of the mastoid facial canal) and other clinical or implant-related risk factors that may contribute to FNS with the occurrence of FNS in patients after CI surgery.

## Material and methods

### Study design and population

This single-center retrospective study was approved by the local institutional review board with a waiver for informed consent. All cone-beam CT (CBCT) examinations were performed after CI surgery (within the first 48 h postoperatively) between January 2016 and October 2018. Patients implanted with perimodiolar electrode CI512 (Cochlear Ltd.) (*n* = 118), lateral wall electrodes FLEX 24 (*n* = 11), and FLEX 28 (*n* = 88) (MED-EL) were included regardless of their age or gender. Two patients with previous history of FNS before the study date were excluded, one with CI512 and other with FLEX 28 electrodes. The final study population consisted of 215 patients with a mean age of 56 ± 18 and a range between 2 and 89 years (males 47.4%, mean age 56 ± 18; females 52.6%, mean age 56 ± 18). According to the occurrence of FNS in 1.5-year follow-up after CI surgery, the included patients were categorized into group 1, patients without FNS (*n* = 205), and group 2, patients with FNS (*n* = 10). In patients with sequential bilateral CI surgery, the ear with the occurrence of FNS was included. In case of negative history of postoperative FNS and sequential bilateral implantation, the first implanted ear was included, and for simultaneous bilateral implantation, the right ear was evaluated.

### Operative and postoperative records evaluation

The operative and the postoperative records of the patients were retrieved from the local database (ORBIS software, Agfa HealthCare). The analyzed data included the CI type and implantation side, date of CI surgery, causes of sensorineural hearing loss (SNHL), and associated diseases/pathologies including previous head trauma, otosclerosis, cholesteatoma, meningitis/encephalitis, mumps, ear inflammation, facial nerve palsy, Meniere’s disease, hearing affection after antibiotic use, and history of postoperative FNS in a follow-up period of 1.5 years after CI surgery.

### Cone-beam CT

All patients were examined using a CBCT device (PLANMECA ProMax-3D Max; Planmeca Oy) with a flat panel detector. Each implanted ear was imaged separately with image acquisition using single 210° rotation and the following scan parameters: 7–11 mA and 120 kV with field of view of diameter (D) 100 × height (H) 90 mm^2^ (for adults) and (D) 85 × (H) 75 mm^2^ (for children), voxel size (0.15 mm, 0.2 mm; isotropic), and focal spot size of 0.6 mm with fixed anode.

### Image evaluation

**Fig. 1:**
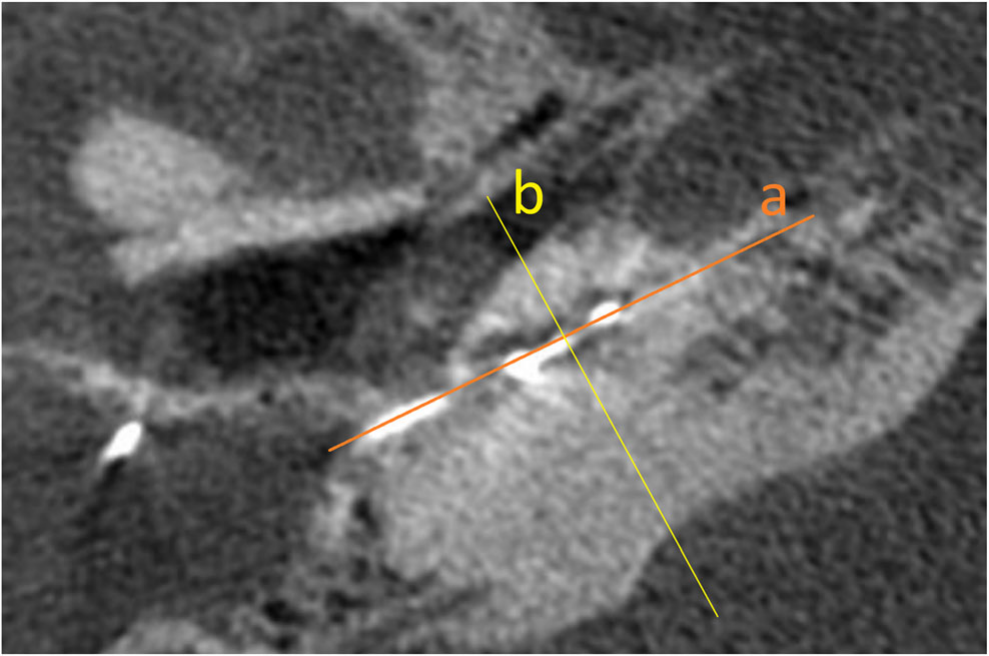
Axial CBCT image shows the reconstruction planes for the different reformatting projections: (a) Cochlear view reformat parallel to the basal turn of the cochlea, (b) Mid-modiolar view reformat perpendicular to the cochlear view plane

**Fig. 2:**
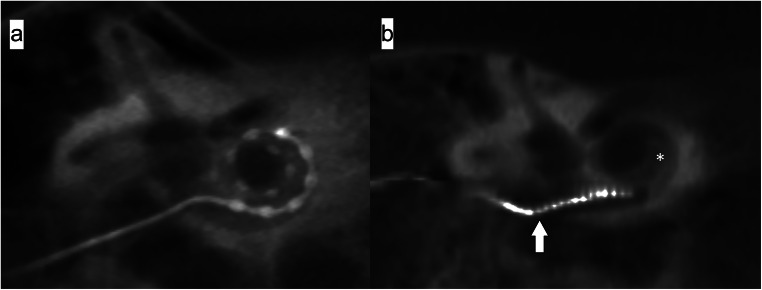
Cochlear view reformats of CBCT images compare the electrode insertion in two patients from both groups: **a** A patient without FNS showing complete insertion of the electrode (FLEX 28-Med-EL; right ear). **b** A patient presenting postoperatively with FNS and an incomplete insertion of the electrode (white arrow) (Contour Advance; Cochlear; right ear); this patient had intracochlear ossification (asterisk)

**Fig. 3:**
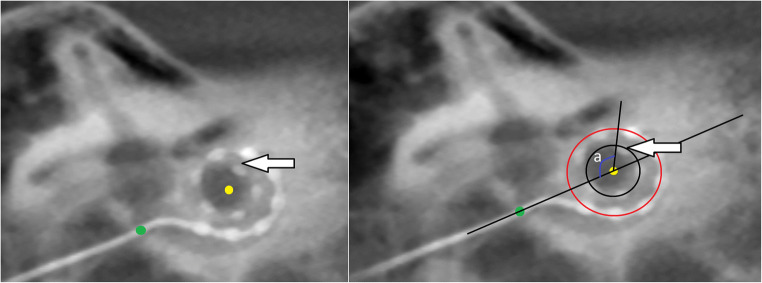
Cochlear view reformat CBCT image shows the measurement of the angle insertion of the electrode inside the cochlea. It equals the numbers of turns the deepest electrode contact (white arrow) forms inside the cochlea around a reference line (the line joining the center of the round window/cochleostomy (green dot) and the center of a circle formed by the 3 most apical electrodes (yellow dot)). The insertion angle measurement = 2 turns - angle (a) = 360° (red circle) + 360° (black circle) - angle (a)
- *Mid-modiolar view*: oblique view in a plane perpendicular to the cochlear view (Fig. 1) for assessing the scalar position of the CI electrodes (Fig. 4).

The obtained images were anonymized and evaluated on certified diagnostic screens (RadiForce RX240; Eizo) using a dedicated PACS viewer (GE Centricity, GE Healthcare) with a window width of 2700 and center of 700. The evaluation of the image datasets was performed in consensus by two experienced head and neck radiologists with 5 years and 10 years of experience blinded to the clinical data of the patients. The following reformatted projections were used:

- *Cochlear view*: oblique coronal reformat parallel to the basal turn of the cochlea (Fig. 1) showing the whole basal turn, round window, oval window, vestibule, superior and lateral semicircular canal, using multiplanar reformations (slice thickness 5mm) for the assessment of extracochlear-electrode-contacts (Fig. 2), and calculation of the insertion angle (Fig. 3) [10].

**Fig. 4:**
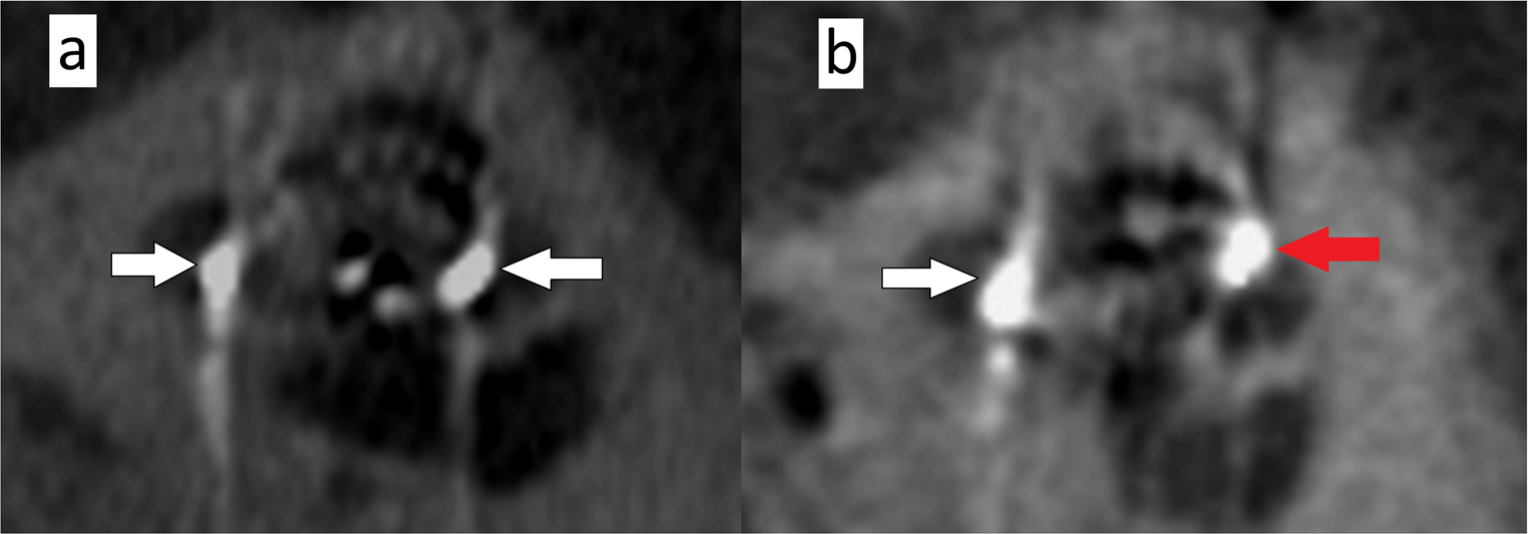
Midmodiolar view reformats of CBCT images: **a** shows the scala tympani insertion of the electrode at the basal turn of the cochlea ( white arrows); **b** shows the shift of the electrode from scala tympani (white arrow) to scala vestibuli (red arrow) inside the cochlea
- *Parasagittal view for the facial recess*: oblique sagittal view obtained in a plane parallel to the superior semicircular canal [11] (Fig. 5):
  - Facial canal wall diameter (mastoid part): defined as the diameter of the facial canal in a plane parallel to the lateral semicircular canal and at the level of the electrode cable in the facial recess.
  - Facial canal wall thickness (mastoid part): defined as the wall thickness of the facial canal in a plane parallel to the lateral semicircular canal and at the level of the electrode cable in the facial recess.
  - Distance between the electrode cable and the facial canal (mastoid part): defined as the distance between the facial canal wall and the electrode cable center in a plane parallel to the lateral semicircular canal.
  - Facial-chorda tympani angle: defined as the angle between the facial nerve and its chorda tympani branch.

**Fig. 5:**
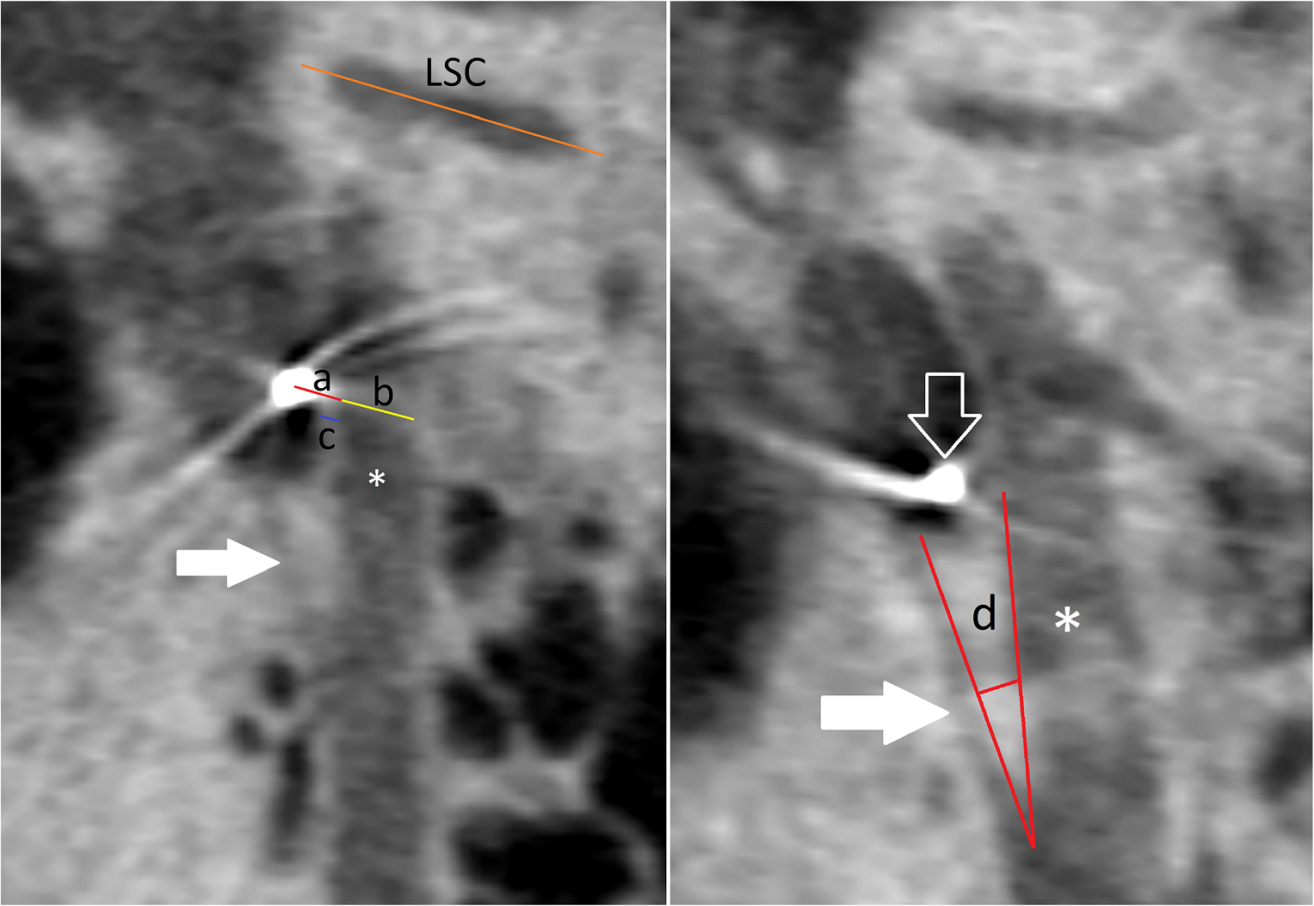
CBCT oblique sagittal reformatted images show measurements of the facial recess region. a: Distance from facial canal (asterisk) to electrode cable (open arrow), b: facial canal diameter, c: facial canal wall thickness, and d: angle between the facial nerve canal and Chorda tympani (white arrow) (LSC, lateral semicircular canal)

### Statistical analysis

Statistical analysis was performed using dedicated software (IBM SPSS Statistics for Windows, Version 27.0). Quantitative data was presented as median with inter-quartile range (IQR) when not normally distributed (according to the Kolmogorov-Smirnov test). For age, additionally mean ± standard deviation (SD) and range were presented. Qualitative variables were presented as number, percentages, and the two independent groups were compared by chi-square test or the Fisher exact test. Comparisons of quantitative data were performed using the Wilcoxon-Mann-Whitney *U* test.

In order to determine the relationship between the predictor variables (mastoid facial canal diameter, its wall thickness and distance to the electrode cable, facial-chorda tympani angle, patients’ age, gender, side of the examined implant, laterality of implants, onset of SNHL, family history of SNHL, revision surgery history, previous history of underlying diseases) and the clinical outcome (FNS occurrence), a univariate logistic regression was conducted. Variables with a significant relationship with the occurrence of FNS (*p* ≤ 0.05) were entered into a multivariable forward logistic regression model to denote the most two significant variables (the first two steps were the included ones because of the low number of cases with FNS). For the resulting odds ratios, 95% confidence intervals were presented. All tests were two-tailed and *p* ≤ 0.05 was considered significant.

## Results

### Descriptive statistics

The included study population was 215 patients which was further classified according to the FNS occurrence into group 1 (without FNS) (*n *= 205, median age (IQR) = 58 (46–71) years) and group 2 (with FNS) (*n *= 10, median age (IQR) = 47 (27–60) years). The different patient-related characteristics are shown in Table 1.

**Table 1:** Patient baseline characteristics

		Group 1 (without FNS) *n* = 205 (%)	Group 2 (with FNS) *n =* 10 (%)	*p* value
Age (years)	Mean ± SD	56 ± 18		
	Range	2–89		
	Median (IQR)	58 (46–71)	41 (27–60)	**0.03**ǂ
Gender	Female	107 (52%)	6 (60%)	0.75*
	Male	98 (48%)	4 (40%)	
Implant type	Perimodiolar	115 (56%)	2 (20%)	0.046*
	Lateral wall	90 (44%)	8 (80%)	
Side examined	Right	117 (57%)	5 (50%)	0.75*
	Left	88 (43%)	5 (50%)	
Implant status	Unilateral	142 (69%)	6 (60%)	0.51*
	Bilateral	63 (31%)	4 (40%)	
Implant surgery	First time	182 (89%)	7 (70%)	0.11*
	Revision	23 (11%)	3 (30%)	
SNHL onset	Adult-onset	128 (72%)	4 (40%)	0.07*
	Early/childhood-onset	50 (28%)	6 (60%)	
	Unknown – onset	27	0	
Family history of SNHL	No	195 (95%)	8 (80%)	0.1*
	Yes	10 (5%)	2 (20%)	

*p *values are written in bold when statistically significant

*FNS*, facial nerve stimulation; *n*, number of patients; *ST*, scala tympani; *SV*, scala vestibuli; *SNHL*, sensorineural hearing loss; *IQR*, interquartile range. *Fisher exact test, ǂWilcoxon-Mann-Whitney *U* test

### Radiological assessment analysis

Two patients were excluded from the facial recess measurements because of the ossified superior semicircular canal due to labyrinthitis ossificans (*n* = 1) and anomalous course of the facial canal (*n* = 1); in both cases, the parasagittal reformatting was not possible. Due to metallic artefacts, the measurements were not possible in some patients as follows: in 5% of patients (*n* = 11) the distance between the electrode cable and the mastoid facial canal as well as the facial canal diameter were not possible, and in 40% of patients (*n* = 86), the facial canal-wall-thickness were not evaluable. The chorda tympani was visualized in 80.9% of patients (*n* = 174) while the plane for measuring the angle between it and the facial nerve was identified clearly in 64.2% of patients (*n* = 138) with a mean = 19.91 ± 0.79. Metallic artefacts also hindered the detection of electrode scalar position in 3% of patients (*n* = 6) in group 1 and 10% in group 2 (*n* = 1). The different measurement results are shown in Table 2.

**Table 2:** Radiological assessment analysis of mastoid facial canal and the insertion of the cochlear implant in the study groups

**Variable**		**Group 1 (without FNS)**	**Group 2 (with FNS)**
Facial canal diameter (mm)	Median (IQR)	1.7 (1.5–1.8)	1.5 (1.4–1.7)
Facial canal wall thickness (mm)	Median (IQR)	0.5 (0.4–0.68)	0.45 (0.1–0.5)
Electrode cable-Facial canal distance (mm)	Median (IQR)	1.4 (1.0–1.7)	1.25 (0.98–1.9)
Facial-Chorda tympani angle	Median (IQR)	19° (14–24.75)	21.5° (15–22.25)
Electrode insertion angle	Median (IQR)	397° (357–540)	540° (367–579)
Variable		Group 1, *n* = 205 (%)	Group 2, *n* = 10 (%)
Extracochlear-electrode-contacts	Present	2 (0.97%)	2 (20%)
Electrode scalar position	ST	156 (76.1%)	4 (40%)
	SV	8 (3.9%)	2 (20%)
	Translocation from ST to SV	34 (16.6%)	2 (20%)
	Translocation from SV to ST	1 (0.4%)	1 (10%)

*FNS*, facial nerve stimulation; *IQR*, interquartile range; *n*, number of patients; *ST*, scala tympani; *SV*, scala vestibuli

### Clinical history analysis

The causes of SNHL and associated diseases in the study groups are shown in Table 3. In group 2, there was one case of inner ear anomaly (incomplete partition type I) associated with anomalous course of facial nerve and history of repeated reimplantation before FNS occurrence, one with Osteopathia Striata with Cranial Sclerosis (OSCS), and another one with Usher syndrome. Three cases with previous meningitis/encephalitis were identified in group 2, one of them had previous facial and abducent nerves palsy following meningitis, and two of them had signs of labyrinthitis ossificans on CBCT. There was one patient suffering with functional left-sided deafness and another one with acute unilateral hearing loss.

**Table 3:** Causes of sensorineural hearing loss and associated diseases in the study population

Previous medical history	Group 1 (without FNS) *n* = 205 (%)	Group 2 (with FNS) *n* = 10 (%)
Head trauma (relevant)	17 (8.3%)	0 (0%)
Meniere’s ds	14 (6.8%)	0 (0%)
Previous ear-surgery	35 (17.1%)	0 (0%)
Cholesteatoma	11 (5.4%)	0 (0%)
Otosclerosis	8 (3.9%)	0 (0%)
Post-meningitis/encephalitis	5 (2.4%)	3 (30%)
Post-mumps	2 (1%)	0 (0%)
Inflammation	7 (3.4%)	0 (0%)
Antibiotic use	5 (2.4%)	0 (0%)
Inner-ear anomalies	0 (0%)	1 (10%)
Facial nerve/chorda tympani injury	4 (2%)	1 (10%)
Other associated diseases/syndromes	26 (12.7%)	2 (20%)

*FNS*, facial nerve stimulation; *n*, number of patients. Subjects could have more than one event

### FNS analysis

For the patients in group 2 (*n* = 10), FNS was reported with immediate onset after CI activation for two patients (20%), within the first year of activation for six patients (60%) and 1 year after activation in two patients (20%) giving a median (IQR) of 6.00 (2.25–10.5) months. One patient with FLEX 28 had mid-array electrode contacts responsible for FNS, but in four patients (three with FLEX 28 and one with Contour Advance), the lower basal electrode contacts were responsible for FNS. Two patients showed extracochlear-electrode-contacts, which were deactivated or adapted in stimulation pulse width (Fig. 3). One patient with FLEX 28 had postoperative dizziness which was cured by deactivating some contacts, but this was followed by FNS (treated by changing the device`s stimulus pattern from biphasic to triphasic). In further four patients, no specific electrode contact could be identified as a cause of FNS and they were treated by pausing or increasing the pulse width of the CI.

### Logistic regression model

The following variables showed significant correlation with FNS occurrence in the univariate logistic regression: patient’s age, previous history of meningitis/encephalitis, early/childhood onset of SNHL, lateral wall electrode, extracochlear-electrode-contacts, scala vestibuli insertion, and the scalar translocation of electrodes. In the final step of the multivariate logistic regression, the history of meningitis/encephalitis and the presence of extracochlear-electrode-contacts were included (Tables 4 and 5).

**Table 4:** Correlation of the FNS occurrence with the other variables using univariate logistic regression

Variable		*p* value
Facial-canal diameter		0.09
Facial-canal-wall thickness		0.27
Cable-facial-canal distance		0.44
Chorda-facial angle		0.75
Age		**0.03**
Gender (female/male)		0.63
Side (left/right)		0.66
Laterality (bilateral/unilateral)		0.54
Onset of SNHL (adulthood/ childhood)		**0.04**
Family history of SNHL		0.06
Previous meningitis/encephalitis history		**0.001**
History of otosclerosis		> 0.99
History of cholesteatoma		> 0.99
History of Meniere’s disease		> 0.99
Previous head-trauma history		> 0.99
Previous antibiotic use history		> 0.99
Previous mumps history		> 0.99
Previous ear inflammation		> 0.99
Previous facial palsy history		0.14
Previous ear-surgery history		> 0.99
Revision surgery history		0.09
Implant type (lateral wall electrode/perimodiolar electrode)		**0.04**
Insertion angle		0.68
Electrode insertion (extracochlear/intracochlear contacts)		**0.002**
Electrode scalar position/ST	SV	**0.02**
	Translocation from ST to SV	0.35
	Translocation from SV to ST	**0.02**

*p *values are written in bold when statistically significant

*SV*, scala vestibuli; *ST*, scala tympani

**Table 5:** Variables with significant *p* values in univariate logistic regression and the forward stepwise logistic regression results (Wald method)

Variable		OR	CI (95%)	*p* value
Univariate logistic regression				
Age		0.96	(0.93–0.996)	0.03
Onset of SNHL (adulthood/ childhood)		0.26	(0.07–0.96)	0.04
Type of implant (lateral wall/ perimodiolar electrode)		5.11	(1.06–24.66)	0.04
Insertion (extracochlear/intracochlear insertion)		25.36	(3.16–203.81)	0.002
Electrode scalar insertion/ ST	SV	9.75	(1.55–61.40)	0.02
	Translocation from SV to ST	39	(2.05–740.77)	0.02
Positive meningitis history		17.14	(3.40–86.42)	0.001
Forward stepwise multivariate logistic regression				
Step (1) Positive meningitis history		13.37	4.73–171.63	< 0.001
Step (2): Positive meningitis history Electrode insertion (extracochlear/intracochlear contacts)		9.981 6.356	3.32–167.45 2.23–611.69	0.002 0.01

*OR*, odds ratio; *CI*, confidence interval; *SV*, scala vestibuli; *ST*, scala tympani

## Discussion

Facial nerve stimulation is one of the common complications following CI surgery [8]. It is often associated with certain conditions as cochlear malformations, otosclerosis, cochlear ossification, and temporal bone fractures [6]. Additionally, postoperative FNS has been linked to the narrow distance between the labyrinthine facial canal and the cochlea [2, 9]. In the current study, the retrospective examination of the clinical data with 1.5-year follow-up revealed FNS occurrence in 4.7% of cases with a median onset of 6 months after CI activation. Smullen et al [12] showed a FNS incidence of 6.5% in their study with a median onset of 3.5 months after CI activation.

In 2016, Diogo et al [11] showed that CBCT can be used to visualize fine anatomical details with low metallic artefacts in postoperative CI patients including the assessment of the mastoid facial canal and the chorda tympani as well as their relation to the electrode cable. We evaluated postoperative CBCT after CI surgery to investigate whether the close anatomical relationship of the electrode cable and the mastoid facial nerve, correlated with the occurrence of postoperative FNS. We assessed the distance between the electrode cable and the mastoid part of the facial canal as well as the facial-chorda tympani angle, assuming that a narrow facial recess may contribute to FNS, but there were no significant correlations. Furthermore, we found no significant correlation between the FNS occurrence and the diameter or the wall thickness of the mastoid facial canal. However, the medians of the facial canal diameter and wall thickness and the distance between the facial canal and the electrode cable were smaller in patients with FNS. Thus, a larger study population may still be needed to confirm these results.

In the current study, the most two significant risk factors correlated with FNS were the radiological detection of extracochlear-electrode-contacts and the previous history of meningitis/encephalitis. These results match prior studies considering extracochlear-electrode-contacts as important risk factor for FNS [6, 12]. Seyyedi et al [3] and Smullen et al [12] reported that the mid-array electrode contacts were predominantly responsible for FNS because of their proximity to the labyrinthine segment of the facial nerve. We found that in four patients with postoperative FNS, the co-stimulation was caused by lower basal electrode contacts which were located near the mastoid segment of the facial nerve. This may indicate that not only the labyrinthine but also the mastoidal course of the facial nerve can be receptive to electric stimulation. Previous history of meningitis/encephalitis was related to the FNS occurrence in some studies which was explained by the facilitation of the electric current propagation from the CI outside the ossified cochlea due to the change in the cochlear bony structure [6, 12, 14]. Similarly, we detected three cases with postoperative FNS who had previous meningitis (two had evident labyrinthitis ossificans).

We encountered an increased occurrence of FNS in cases with scala vestibuli insertion, and scalar translocations rather than scala tympani insertion, while the electrode’s insertion angle had no potential correlation with FNS. FNS was also more notable with the straight electrodes as compared with perimodiolar electrodes. These findings are consistent with the results of Seyyedi et al [3] and Battmer et al [13] which can be explained by the fact that the lateral wall electrodes may require higher stimulation thresholds than perimodiolar ones or their closer location to the facial nerve. On the other hand, Smullen et al [12] reported no potential difference between diverse electrode types regarding FNS, but they recommended perimodiolar electrodes, because these could cause FNS only at significantly high stimulation levels.

In the study population, none of the eight patients diagnosed with otosclerosis presented with FNS which was concordant with the results of Seeman et al [15]. However, there were other studies that identified otosclerosis as a risk factor [3, 16, 17]. This discrepancy could be explained by the fact that our otosclerosis patients have been implanted with perimodiolar electrodes, which may have reduced FNS occurrence by lower stimulation threshold and their longer distance to the facial nerve course.

By examining the clinical data of our patients, FNS occurrence was more noticed in younger patients, which was concordant with the results of Cushing et al [6] who reported an FNS incidence of 31–78% in children. In addition, FNS was more encountered in cases with early/childhood onset of SNHL rather than those with adulthood onset of SNHL but we found no correlation between the positive family history of SNHL and postoperative FNS occurrence .

Although some studies considered previous temporal bone fractures as a risk factor for FNS, none of our patients with previous history of head trauma showed FNS which was in agreement with the results of Lachowtska et al [5, 18]. FNS has also been reported in patients with cochleo-vestibular anomalies [6, 7]. We examined one case of incomplete partition type I associated with facial nerve anomalous course that had previously repeated reimplantation and suffered lately from FNS. We also had two cases with syndromic hearing loss including Usher syndrome (which was also reported in Smullen et al [12] in one case with post-CI FNS) and osteopathia striata with cranial sclerosis who suffered post-CI FNS; the latter could be related to the change in the properties of skull bones which facilitated the electric current propagation. Although 30% of the evaluated patients suffering from FNS had CI revision surgeries before the occurrence of FNS (20% with repeated surgeries), there was no significant correlation between the previous re-implantation history and FNS occurrence.

The results of our study should be interpreted in the context of the study design and consequent limitations. The low number of patients with FNS, concomitant with the reported low incidence of FNS after CI surgery, may limit the general applicability of the study results; thus, larger studies are warranted to confirm our results. Although CBCT imaging is supposed to have relatively lower metallic artefacts and higher spatial resolution compared with multidetector CT, the metallic artefacts induced by CI limited the radiologic assessments in a non-negligible number of patients [19, 20]. This might have lowered the statistical power of a part of the study, so the use of new metal artifact-suppressing algorithms in CBCT imaging in the forthcoming research might be helpful.

In conclusion, our data suggest that the radiological assessment of the mastoid facial canal and its positional relationship to the CI electrode in the facial recess are not predictors for the occurrence of postoperative FNS. However, radiologically detectable extracochlear-electrode-contacts and previous history of meningitis/encephalitis may be regarded as the most two important predictors. The deviations from the ideal intracochlear electrode location in scala tympani, the lateral-wall-CI type, the younger patient’s age, and early onset of SNHL may be also considered important risk factors for postoperative FNS.

## Declarations

### Guarantor

The scientific guarantor of this publication is Prof. Dr. Thomas J. Vogl.

### Statistics and biometry

One of the authors has significant statistical expertise.

### Methodology

• Retrospective

• case-control study

• Performed at one institution

## Abbreviations

CBCT: Cone-beam computed tomography
CI: Cochlear implant
FNS: Facial nerve stimulation
SNHL: Sensorineural hearing loss
